# Molecular Evolution and Characterization of Hemagglutinin (H) in Peste des Petits Ruminants Virus

**DOI:** 10.1371/journal.pone.0152587

**Published:** 2016-04-01

**Authors:** Zhongxiang Liang, Ruyi Yuan, Lei Chen, Xueliang Zhu, Yongxi Dou

**Affiliations:** State Key Laboratory of Veterinary Etiological Biology, Key Laboratory of Epizootic Diseases of Grazing Animals of Ministry of Agriculture, Lanzhou Veterinary Research Institute, CAAS, Lanzhou, Gansu, China; National Institute of Health, ITALY

## Abstract

Peste des Petits Ruminants (PPR) is an acute, highly contagious, and febrile viral disease that affects both domestic and wild small ruminants. The disease has become a major obstacle to the development of sustainable Agriculture. Hemagglutinin (H), the envelope glycoprotein of Peste des Petits Ruminants Virus (PPRV), plays a crucial role in regulating viral adsorption and entry, thus determining pathogenicity, and release of newly produced viral particles. In order to accurately understand the epidemic of the disease and the interactions between the virus and host, we launch the work. Here, we examined *H* gene from all four lineages of the PPRV to investigate evolutionary and epidemiologic dynamics of PPRV by the Bayesian method. In addition, we predicted positive selection sites due to selective pressures. Finally, we studied the interaction between H protein and SLAM receptor based on homology model of the complex. Phylogenetic analysis suggested that *H* gene can also be used to investigate evolutionary and epidemiologic dynamics of PPRV. Positive selection analysis identified four positive selection sites in *H* gene, in which only one common site (aa246) was detected by two methods, suggesting strong operation structural and/or functional constraint of changes on the H protein. This target site may be of interest for future mutagenesis studies. The results of homology modeling showed PPRVHv-shSLAM binding interface and MVH-maSLAM binding interface were consistent, wherein the groove in the B4 blade and B5 of the head domain of PPRVHv bound to the AGFCC′ β-sheets of the membrane-distal ectodomain of shSLAM. The binding regions could provide insight on the nature of the protein for epitope vaccine design, novel drug discovery, and rational drug design against PPRV.

## Introduction

Peste des Petits Ruminants (PPR) is an acute, highly contagious, and febrile viral disease that affects both domestic and wild small ruminants. Clinically, PPR infection is characterized by leukopenia, pyrexia, congestion of mucosal surfaces, ocular and nasal discharge, erosive stomatitis, diarrhea, and suppression of the immune system often leading to co-infection[1–3]. The disease is considered to be a big “dam” to the development of sustainable agriculture across the developing countries as a notifiable disease listed and has recently been oriented by the World Organization for Animal Health (OIE) and the Food and Agriculture Organization (FAO) for eradication with the aim of global elimination of the disease by 2030 [4]. The causative agent of the disease, Peste des Petits Ruminants Virus (PPRV), is an enveloped virus with a non-segmented, negative-strand RNA genome, and is classified as a member of the genus *Morbillivirus* along with Measles virus (MV) and Rinderpest virus (RPV) [2]. The virus genome length is approximately 16,000 nucleotides (nt) and encodes six essential structural protein such as H and fusion (F) proteins and the two non-structural proteins[5–7]. Unusually noticeably, H and F are two kinds of glycoproteins of the virus. To infect cells, enveloped viruses must interact with their receptors on host cells for enveloped proteins and induce fusion of the viral membrane with the host cell membrane in order to enter host cells. Dramatically, H protein is responsible for attaching the virions to the host receptors and generally regulates viral adsorption and entry, determining pathogenicity, and releasing newly-produced viral particles [8, 9]. In addition, neutralizing antibodies against H protein serve as protective antibodies in PPRV infection. Multiple researches have shown the epitopes for the neutralizing antibodies and T cell determinants against H protein [10–12]. Further analysis of H protein will further our understanding of the molecular evolution of the virus and the presence of host-specific mechanisms, which will be significant in the control and elimination of the PPR disease.

Phylogenetically, PPRV can be divided into four genetically distinct lineages, I-IV, based on the partial region of *N* or *F* gene [13, 14]. The lineages are associated with the geographic distribution of the virus, with lineages I and II exclusively isolated in West Africa, lineage III in Arabia, East Africa, and southern India, and lineage IV in southern Asia, the Middle East, and more recently, northern Africa [2,4]. Herein, we rebuild the phylogenetic tree of the *H* gene to estimate the evolutionary relationships of various strains in different regions or dates. This analysis will enable a more precise evolutionary and phylogenetic assessment of the relationships among the four PPRV genetic lineages.

As a result of the selection pressures of the host immune system, antigenic changes may occur through positively-selected amino acid substitutions [15–17]. The selective pressures could lead to a change in the antigenic epitopes and may cause a change in the receptor between the host and virus. Indeed, attachment glycoprotein of an essential antigen of respiratory syncytial virus and parainfluenza virus could show frequent positive selections in the antigenic epitopes of the protein [18, 19]. Recently, Muniraju M. evaluated the mean ratios of Nonsynonymous (dN) to Synonymous (dS) substitutions per site of concatenated coding regions of the PPRV genome, which indicated there may be positive selections sites in the coding region of *H* gene [20]. However, codon sites under positive selection are not known. Thus, it may be important to analyze the molecular evolution of *H* gene in PPRV. We estimated positive selection sites in the genes by the CODEML program of PAMLX [21]. Furthermore, single likelihood ancestor counting (SLAC), fixed-effect likelihood (FEL), internal fixed-effect likelihood (IFEL), and random effect likelihood (REL) method in the Date monkey (http://www.datamonkey.org) were used to evaluate the result. We used the likelihood method with more effective models of evolution to analyze our collected data [22].

A *Morbillivirus* initially infects a broad range of immune system cells and then spreads to epithelial cells. However, SLAM (signaling lymphocyte activation molecules; also known as CDw150), which appear on the surface of activated T and B lymphocytes, macrophages, and dendritic cells, acts as a receptor for all morbilliviruses, including PPRV [23–26]. Recent reports determined that the interaction of SLAM with MV was not only the first step of the virus invading the host, but also the important cause of the pathological changes in the host organism and presentation of clinical symptoms [26]. Because of this background, some researchers have explored the interaction between SLAM and PPRV, and recent evidence demonstrated the inference that SLAM serves as the cell receptor for H protein in PPRV [24,25]. However, there is inconclusive evidence that would reveal more detailed data of the interaction, such as domains or motifs of the protein-protein interaction. As we all known, the small segments of antigen may be the antigenic determinants or the epitopes that are limited in eliciting the preferred immune response. We hope to find the antigen epitopes that have the ability to produce neutralizing antibody with the combination of H and SLAM in response through to understand H protein interaction with SLAM in the three-dimensional (3D) structure of protein. It is well known that the 3D structure of a protein determines its function. Above all, the knowledge about the 3D structure of protein is extremely important for epitopes vaccine design, novel drug discovery, and rational drug design. The 3D structural model of the H protein isn't yet known. Nevertheless, the structure of MV H (the highly homologous protein of PPRV H) was reported by Christopher’s and Yusuke’s groups in 2007 [27, 28], which have provided the greatest aid in studying 3D structure of PPRV H. Therefore, in our study, we used homology modeling to build the structural model of H protein. In order to understand H protein-SLAM interaction, the 3D model of H-SLAM complex was predicted by homology modeling using Discovery Studio (DS) v4.5 (Accelry, San Diego).

Hence, our study is of significance to the future exploration of molecular evolution and characterization of H protein, which may provide useful insights in studying molecular epidemiology of the virus and in predicting epitopes for vaccine design against PPR.

## Materials and Methods

### Sequence Collection and Multiple Sequences Alignment (MSA)

Biochemically characterized *H* gene of PPRV (Nigeria/75/1 strain, GenBank accession No.X74443) as query sequence was obtained from the National Center for Biotechnology Information (http://www.ncbi.nih.gov/) database. The full-length coding sequence of *H* gene for available PPRV strains from the disease-endemic countries were downloaded from GenBank at NCBI using inference sequence to search by BLASTn against nucleotide collection (nr/nt) database with default parameters. We concentrated an integrated collection of the full-length coding sequences for *H* gene of PPRV. Sequences with 100% identity and suspicious were excluded from the dataset in further analyses. Additionally, the outgroup, RPV (Kabete O strain, GenBank accession No.X98291; RBOK strain, GenBank accession No.Z30697), was added to the dataset. Multiple sequences alignment (MSA) was the critical step in phylogenetic analysis and the alignment played a crucial role in extracting evolutionary information from a large number of sequences. In this study, MSAs for coding sequences of *H* gene were performed by MUSCLE [29] with the algorithm using default parameters in the software Molecular Evolutionary Genetics Analysis (MEGA) 6.0.6 (http://www.megasoftware.net/) [30]. The result of test of substitution saturation performed on all sites using DAMBE5 [31] showed that the observed Iss is significantly lower than Iss.c (p = 0.0000), and the estimated Transition/Transversion bias (R) is 4.44. After processing, 36 strains of PPRV were identified in this study, which are shown in Table 1.

**Table 1:** Reportorial *H* gene in Peste des Petits Ruminants Virus.

Lineage a	Year b	Region c	Accession Number	Strains Abbreviation
IV	2007	China Tibet	GQ184301	Chi200701
IV	2007	China Tibet	FJ905304	Chi200702
IV	2008	China Tibet	JX217850	Chi2008
IV	2013	China XJYL	KM091959	Chi2013
IV	2014	China HNZM	KM089832	Chi201401
IV	2014	China GDDG	KP868655	Chi201402
IV	2014	China JL	KM816619	Chi201403
IV	2014	China HNNY	KM089830	Chi201404
IV	2014	China BJ	KP260624	Chi201405
IV	2014	China HNZK	KM089831	Chi201406
IV	2010	Ethiopia	KJ867541	Eth2010
IV	1994	India Izatnagar	KR140086	Ind199401
IV	1994	India Izatnagar	KF752443	Ind199402
IV	1996	India Sungri	AJ512718	Ind199601
IV	1996	India Sungri	KJ867542	Ind199602
IV	1996	India Sungri	GQ452016	Ind199603
IV	1996	India Sungri	AY560591	Ind199604
IV	1996	India Sungri	KF727981	Ind199605
IV	2003	India Jhansi	GU014573	Ind200301
IV	2003	India Jhansi	EU344741	Ind200302
IV	2003	India Bhopal	FJ750563	Ind200303
IV	2014	India TN	KR261605	Ind201401
IV	2014	India TN	KT270355	Ind201402
IV	2008	Morocco	KC594074	Mor2008
IV	2011	Iraq Kurdistan	KF648288	Ira2011
IV	2000	Turkey	AJ849636	Tur2000
IV	2011	Turkey Afyon	KJ914667	Tur2011
III	1994	Ethiopia	KJ867540	Eth1994
III	1983	Oman	KJ867544	Oma1983
III	2011	Kenya	KM463083	Ken2011
III	1986	UAE	KJ867545	UAE1986
III	2012	Uganda	KJ867543	Uga2012
II	1969	Benin	KR781450	Ben1969
II	2011	Benin	KR781449	Ben2011
II	2009	Cote d'Ivoire	KR781451	CIV2009
II	2010	Ghana NK	KJ466104	Gha2010
II	1975	Nigeria	X74443	Nig197501
II	1975	Nigeria	HQ197753	Nig197502
II	1976	Nigeria	EU267274	Nig1976
II	2013	Senegal	KM212177	Sen2013
I	1989	Cote d'Ivoire	EU267273	CIV1989
I	1969	Senegal	KP789375	Sen1969

^a.^ Lineages of isolates of PPRV were named by following the classification of lineages based on partial *N* gene sequence phylogenetic analysis.

^b.^ Collection date.

^c.^ Region isolated.

### Phylogenetic Analysis

Bayesian tree was reconstructed based on codon positions using all available sequences we obtained to estimate the evolutionary relationship by TOPALi v2.5 [32]. Prior to tree construction, the best-fitting evolution model GTR+I+G was selected Akaike Information Chriterion (AIC) in Modeltest v3.7 (June 2005) and MrMTgui (Nuin, 2007) [33] The Bayesian tree was rebuilt using the following settings: 4 runs, 1,000,000 generations, 100 of sample frequency and 25% burn in. To confirm the topology of the Bayesian tree, Maximum Likelihood (ML) tree was rebuilt using the HIVw+G+F model, which was selected using Akaike Information Chriterion (AIC) in ProtTest 2.4 [34]. And the ML tree topology was optimized using NNI method and a BioNJ starting tree [35] and 1000 Bootstrap replicates were used to estimate the reliability of the internal nodes. Bayesian and ML, the different methods, were used to reconstruct phylogenetic tree to ensure the reliability of the tree.

### Selective Pressures Analysis

To test positive selection in individual codons of the *H* gene, ω ratios were compared using the two ML frameworks, the CODEML program of PAML X software package and the Hyphy package implemented in the Data Monkey Web Server.

In CODEML, site model was selected to detected positive selection. Three pairs of models, Model0 (one ratio) and Model3 (discrete), Model1 (nearly neutral) and Model2 (positive selection), Model7 (β) and Model8 (β & ω), were performed to evaluate our collected data set. M0 *versus* M3 was used to test for rate heterogeneity among amino acid sites; M8 *versus* M7 and M1 *versus* M2 were used to determine the possible sites under selection. LRTs were performed to test the positive selection sites by comparing the nested models [36]. The Bayes empirical Bayes (BEB) analysis and the Naive Empirical Bayes (NEB) analysis in the case of comparing models was used to calculate the Bayesian posterior probabilities (BPP) of the codon sites under positive selection[37, 38]. In this test, the *H* gene tree was employed as the guide tree. In order to further determine the positively selected sites acquired in the CODEML, a battery of ML methods were performed in the Data Monkey Web Server. The automatic model selection tool on the server was used to research the best fitting nucleotide substitution models. Four different codon-based maximum likelihood methods, SLAC, FEL, REL and IFEL were used estimate the dN/dS (also known as Ka/Ks or ω) ratio at every codon in the alignment of all available sequences[39]. The significance level of *p*-values (*p* < 0.05) for SLAC, FEL, and IFEL was used to determine codons under positive selection and accepted sites, and Bayes Factor >50 for REL as candidates for positive selection sites.

### Homology Modeling

The monadelphous structural model of H protein and PPRVHv-shSLAM (the H protein of PPRV vaccine strain and the sheep SLAM) complex structural model were generated by Homology modeling in the DS v4.5. The sequence of Hv (the H protein of PPRV vaccine strain, GenBank accession No.CAJ01700) used as target sequence to build monadelphous model. The coupling of Hv and shSLAM (GenBank accession No.NP_001035378) were used as target sequence to build a compound model. In terms of the refined model, the Accelrys CHARMm force field was used for the simulation, and Ramachandran plot was selected for the validation[40]. The structures were optimized by Generalized Born (GB) implicit-solvent model[41]. Energy minimization of the selected model was performed *via* the Steepest Descent method (5000 steps). The model structure was refined using a Conjugate Gradient method (2000 steps). For a better understanding of the protein-ligand interactions, three complexes were generated and the DOPE scoring function and PDF Total Energy were used for score calculation for selecting best complex model. Otherwise, Calculate Interaction Energy (Binding) was selected to analyze for binding interaction and binding energy using the following settings: Permittivity = 75 F/m and Nonbond List Radius = 14.0, Nonbond Higher Cutoff Distance = 12.0 and Nonbond Lower Cutoff Distance = 10.0. Thereafter, alanine-scanning mutagenesis for binding interface was used to evaluate the affinity of protein-ligand. Figures were produced using DS v4.5 Client.

## Results

### Phylogenetic Analysis

**Fig 1:**
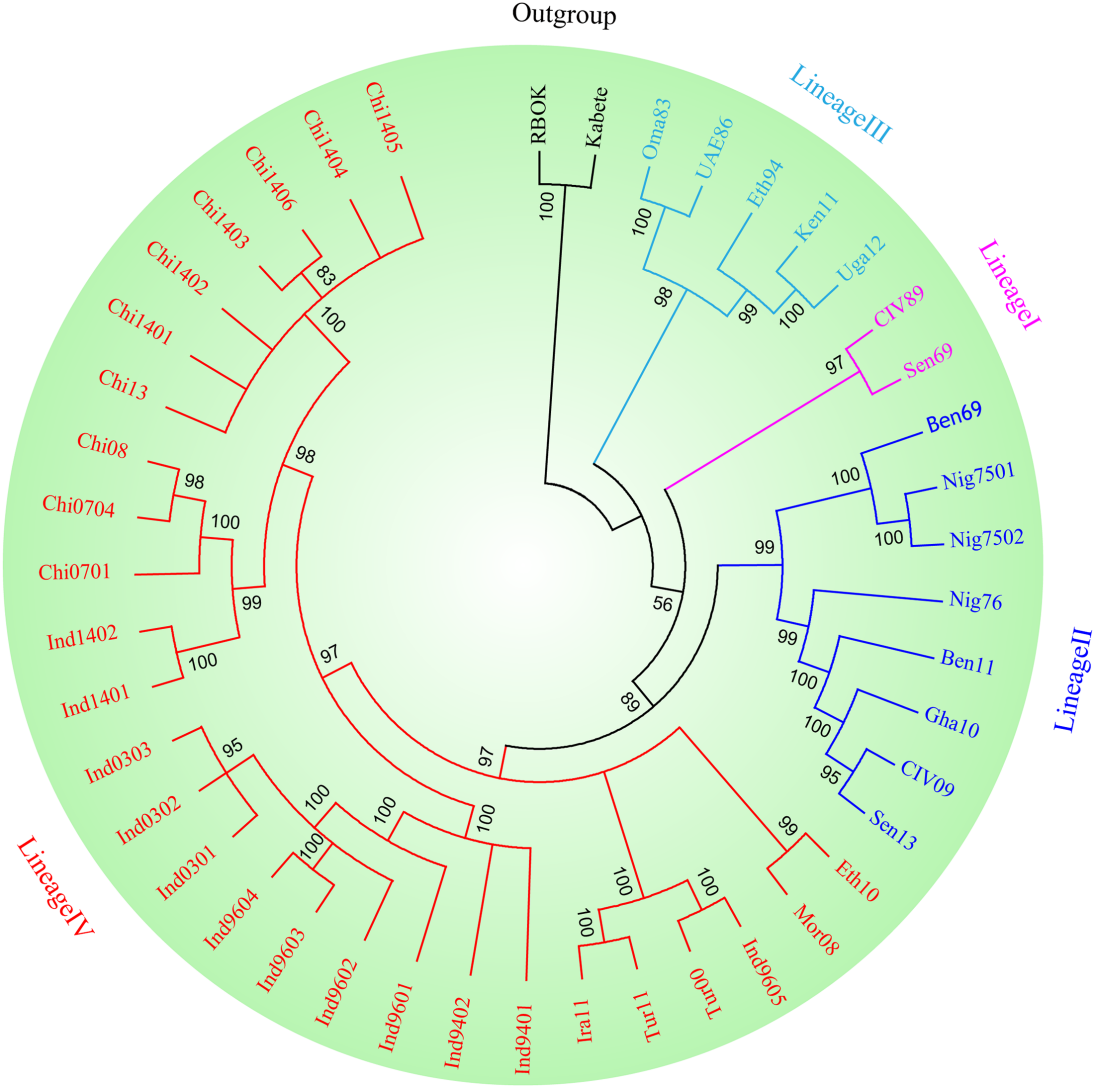
Phylogenetic tree of PPRV based on the hemagglutinin (*H*) gene. The tree was constructed using GTR+I+G model by the Bayesian method. The topology patterns of the phylogenetic tree shows all strains included in four lineages. Phylogenetic analyses were performed using the TOPALi v2.5 package.

Phylogenetic analysis demonstrated that the topology patterns of the phylogenetic tree included four clades, and dendograms with highly coincident was constructed in two cases (Bayesian and ML trees). Obviously, PPRV could be divided into four lineages based on *H* gene. The isolates from India, China, Ethiopia, Morocco, Turkey, and Kurdistan belonged to lineage IV and were confined in a big clade in the phylogenetic tree, which could be further branched into four minor clades. UAE86 strain, Oma83 strain, Eth94 strain, Uga12 strain and Ken11 strain belonged to lineage III. The strains from Nigeria and Gha10 strain, Sen13 strain, and CIV09 strain were confined in a clade, which belonged to lineage II. And Sen69 and CIV89 strains belonged to lineage I (Fig 1).

### Selective Pressures Analysis

In the PAML test, phylogenetic tree was utilized to detect the positive selection, and the estimated parameters of different models and the LRT results are provided in Table 2. M0 indicated that *H* gene underwent the purifying selection (ω < 1, Table 2) and very short regions or on only a few sites underwent positive selection. LRT (-2ΔlnL = 67.9324) of M0 *vs* M3 was performed, and the results (*p* < 0.001) showed the presence of selection pressure at codon positions. One site (aa246) could be underwent positive selection with a posterior probability larger than 0.99 by BEB and NEB. But, there were no sites identified as being under went positive selection with the posterior probability larger than 0.95 by BEB and NEB. However, the results of the LRT test statistic of M7–M8 comparison was 4.1982 (*p* < 0.05). M7 is rejected in favor of M8revealing that the model which allowed positive selection better than that which did not allowed positive selection. Additionally, the BEB and NEB approaches detected one site (aa246) under positive selection with BPP values larger than 0.95.

**Table 2:** Likelihood values and parameter estimates for the *H* gene in PPRV.

Model	np^a^	-ln L	Estimates of parameters			*P*-value	PSS^b^
M0 (one-ratio)	72	7368.0629	ω = 0.2087				--
M3 (discrete)	76	7334.0967	p0 = 0.4579	p1 = 0.5303	p2 = 0.0117	*P* < 0.001	aa246**
			ω0 = 0.0039	ω1 = 0.3535	ω2 = 2.0661		
M1 (Nearly Neutral)	73	7338.8989	p0 = 0.8883	p1 = 0.1118			--
			ω0 = 0.1254	ω1 = 1.0000			
M2 (Positive Selection)	75	7338.8989	p0 = 0.8883	p1 = 0.0543	p2 = 0.0573	NS	
			ω0 = 0.1253	ω1 = 1.0000	ω2 = 1.0000		
M7 (β)	73	7336.5503	p = 0.4194	q = 1.5364			--
M8 (β & ω)	75	7334.4513	p0 = 0.9946	p1 = 0.0053	p = 0.5113	*P* < 0.05	aa246*
			q = 1.9956	ω = 2.6148			

^a.^ Number of parameters.

^b.^Positive-selection sites (PSS) are inferred at posterior probabilities > 0.95. The lists of sites are identical between Naive Empirical Bayes (NEB) and Bayes Empirical Bayes (BEB) analysis in M2 and M8, while only Naive Empirical Bayes (NEB) analysis was used in M3.

*: P>95%;

**: P>99%

To confirm the test, we estimated positive selection sites in the strains using SLAC, FEL, IFEL, and REL methods in the Data Monkey. Detailed data are shown in Table 3. Results showed that aa246 underwent positive selection by the SLAC method test; three sites (aa246, aa419, and aa574) underwent positive selection by the FEL and IFEL method test; two sites (aa176 and aa246) underwent positive selection by the REL method test. The common site of positive selection estimated by four methods was aa246 which was also determined by PAMLX.

**Table 3:** Phylogenetic tests of positive selection in the *H* gene.

Model	Positive selection sites	Significant Level
		p-value ^a^
SLAC	aa246	0.0110
FEL	aa246	0.0035
	aa419	0.0367
	aa574	0.0449
IFEL	aa246	0.0077
	aa419	0.0497
	aa574	0.0147
		BF ^b^
REL	aa176	296.2030
	aa246	1467.6100

^a.^ Codons with *P* values <0.05.

^b.^ Codons with Bayes factor >50.

### Homology Modeling

**Fig 2:**
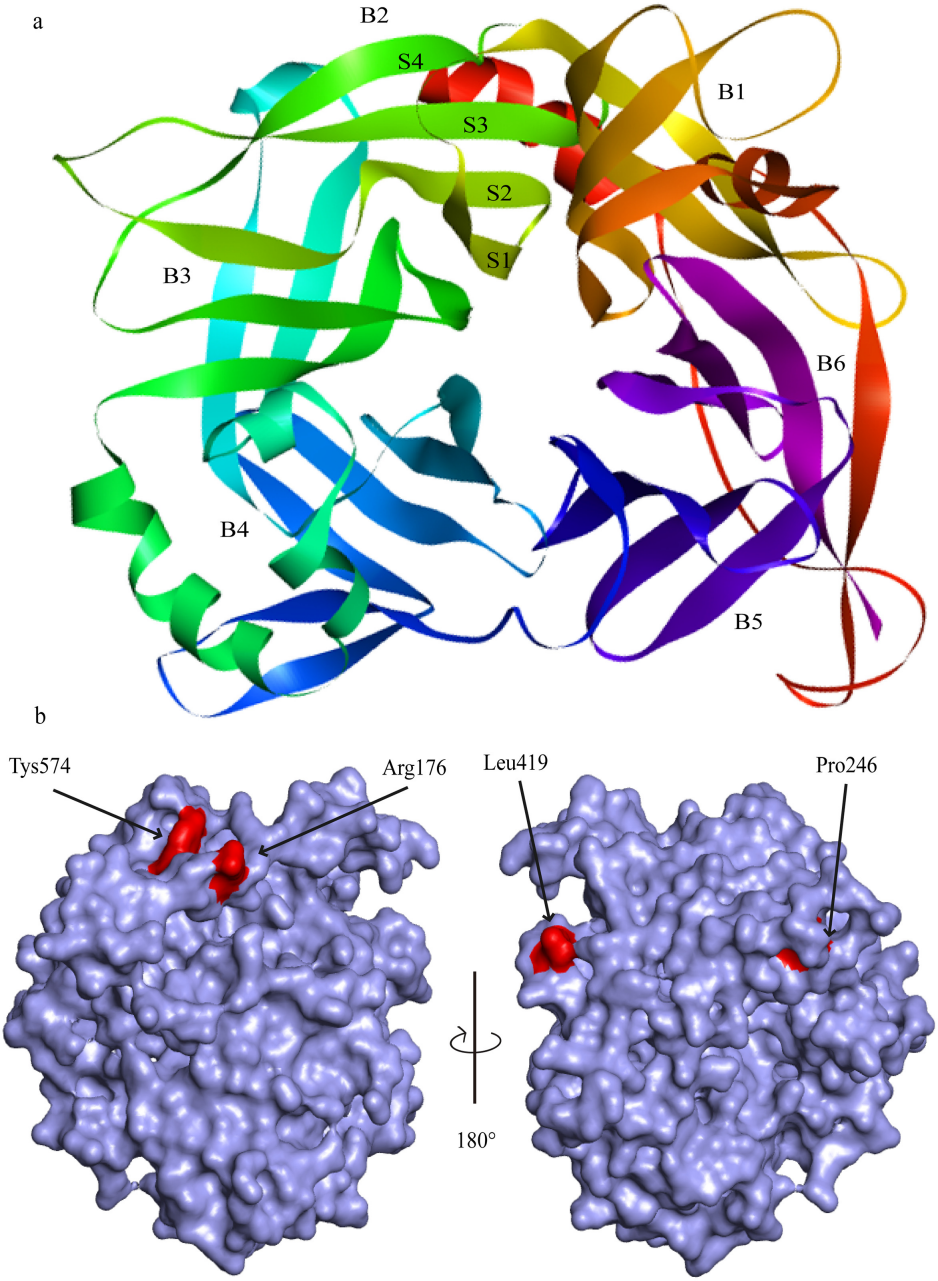
Cartoon and Solvent drawing of predicted PPRV Hv. (A) The fold of H is a β-propeller with six blades (B1~B6) surrounding a large cavity. Every one of blade contains four anti-parallel β-strands (S1–S4), and the six blades are connected through the loops between S1 of the next and S4 of one module. (B) Mapping of the positive selection sites on the H protein structure.

**Fig 3:**
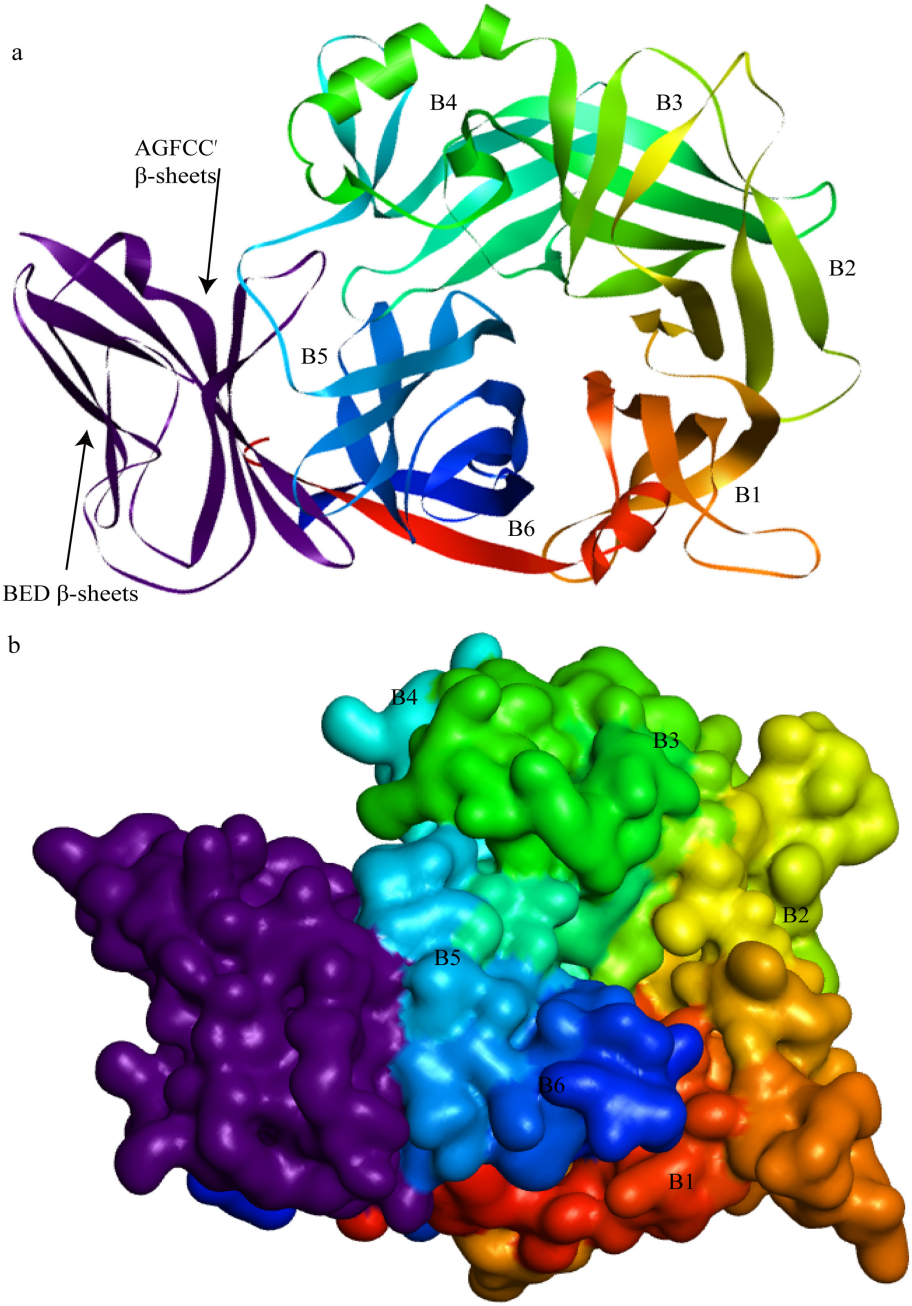
Cartoon and Solvent drawing of predicted PPRVHv-shSLAM complex. (A)The PPRVH head domain exhibits the six-bladed β-propeller fold (rainbow colors) and forms a monomer. The sheep SLAM (purple) exhibits a typical β-sandwich structure with BED and AGFCC′β-sheets. The groove in the B4 blade and B5 of the head domain of PPRVH bind to the AGFCC′ β-sheets of the membrane-distal ectodomain of shSLAM. (B) The pattern of the PPRV with SLAM.

Based upon blast results, MV H (PDB ID:2ZB5) was considered as an ideal homologue and used as a template for homology modeling as both viruses belong to the same genus (genus *Morbillivirus*); therefore, the MVH-maSLAM complex (the MV H-marmoset SLAM complex, PDB ID:3ALZ) was used as a template for complex homology modeling. The result of building model for Hv showed that the fold of H monomer was a β-propeller with six blades (B1∼B6) surrounding a large cavity. Every one of blade contained four antiparallel β-strands (S1–S4), and the six blades were connected through the loops between S1 of the next and S4 of one module (Fig 2a) The Ramachandran plot (φ/ψ) distribution of the backbone conformation angles for each of the residues of the refined structure revealed that 91.9% and 7.8% were in the favored region and allowed region, respectively. In predicted PPRVHv-shSLAM complex by homology modeling, the PPRVH head domain exhibited the six-bladed β-propeller fold (rainbow colors) and formed a monomer. SLAM-V (purple) exhibited a typical β-sandwich structure with two β-sheets: BED and AGFCC′ (Fig 3). In addition, we compared the interface residues (Table 4) of PPRVHv-shSLAM and MVH-maSLAM. To verify the contributions of the selected residues on protein-ligand complex, we designed a series of mutants for both proteins and evaluated their affinity. The residues in protein-ligand binding interface were respectively selected for alanine scanning. In the interface of PPRVHv-shSLAM complex, the mutation energy of Phe552, Arg533, Arg191, Tyr553, Tyr543, Arg503, Asp505, and Pro554 on PPRVHv (S1 Table) and Lys78, Lys76, His130, His62, Leu92, Leu64, Val128, and Glu123 on shSLAM (S2 Table) were more than 1.00 kcal.

**Table 4:** Interface residues comparison between PPRVHv-shSLAM and MVH-maSLAM complex.

Name	PPRVHv-shSLAM				MVH-maSLAM			
**H-Bond**	R191:S132	R191:S50	T192:H130	T194:V128	T192:R130	I194:V128	R533:E123	Y543:V74
	T194:V128	R195:S127	R195:S127	A196:V126	G196:I126	R533:E123	R556:N125	D507:K77
	L483:D73	R533:E123	Y553:V126	R556:E124	D507:K77	I194:V128	T192:R130	T193:V128
	L189:S32	S550:K76	S550:K76	D530:K78	R195:I126	R195:I126	R533:E123	D505:K77
	D530:K78	S532:K78	T194:V128	T192:H130				
**Salt Bridge**	D507:K78	D505:K78			K78:D507	K78:D505	K78:D505	E123:R533
					E123:R533			
**Pi Interaction**	P552:H130	P552:H130	Y553:V128	R533:H62	R533:H61	R533:H61	F552:V63	Y553:V128
					P191:F131			

## Discussion

In this study, we utilized *H* gene of available PPRV strains, which were divided into four lineages based on *N* or *F* gene from different endemic countries, to estimate the molecular evolution of the virus and analyzed the interaction between the virus and host.

It well known that PPRV can be divided into four genetically distinct lineages (I-IV). Historically, isolates from Africa were numbered lineage I-III according to the spread of the virus from West Africa to East Africa[14, 42]. Keeping to this viewpoint, West African isolates from Senegal and Ivory Coast belonged to lineage I; the strains derived from Ghana and Nigeria were formed lineage II; and the strains detected in Ethiopia, UAE and Oman were formed lineage III. Because there was no effective action to control PPR in the past for a period of time, the prevalence of the disease has become more complicated. There was the cross of the lineage in different areas. PPRV of lineage IV appear constantly in the Arabian Peninsula, the Middle East, Southern Asia, and across several African territories, and still in rapid spread [2]. Since PPR emerged for the first time in the Tibet autonomous region of China in 2007, the disease has been reported in China. Because of some effective measures were taken, the epidemic was relatively stable. A study about lineage 4 has suggested that the *N* gene is more divergent and therefore more suitable for phylogenetic distinction between closely related circulating viruses[43]. However, H protein is a membrane glycoprotein of PPRV and very conservative. It may provide useful insights on the epidemic of PPR to study epidemiology of PPRV based on *H* gene. Following our research, the phylogenetic analysis on the basis of *H* gene showed highly similar evolutionary relationship contained in four clusters of lineages. Thus, *H* gene could also be used to analyze the evolutionary relationships of different isolates. Therefore, the evolutionary relationship of different isolates based on *H* gene has a guiding significance in epidemiology.

In the longest co-evolution between viruses and host, molecular adaptations that optimize the organism’s survival in a given living environment can be accumulated and inherited. To probe deeply into how that evolutionary power drives protein evolution and how gene sequences differ in the various strains or species, phylogenetic analysis by maximum likelihood was selected to detect selection or adaptation in this study. The M0 model (ω = 0.206, ω < 1) demonstrated that the entire *H* gene underwent the purifying selection, indicating that overall *H* gene was relatively conserved. This might be correlated with the functionalism and structuralism of H protein which mediates the virus binding to host cellular receptors, a first step in the progression of PPRV infection. However, a study by Yang, Z *et al*. pointed out that the average ω ratio for overall sequence was very seldom greater than 1 and the positive selection seldom happened in some structural domains[44]. In our study, some positive selection sites were found; and to the best of our knowledge, this is the first study that reports this finding in PPRV H. Recently, Kimura H. *et al*. reported that there were 8 positive selection sites in 297 strains of *H* gene of congeneric virus (MV) in the prevalent Asian genotypes, D3, D5, D9, and H1 [45]. Nevertheless, a previous report showed that MV in 50 strains of the *H* gene of the various genotypes had 14 positive selection sites [46]. Our research about the *H* genes of PPRV indicated that four lineages had one positive selection site (*p* < 0.05) by BEB analysis. However, four positive selection sites were estimated using the HyPhy package. In the previous report, Sinnathamby *et al*. used autologous skin fibroblast cells to identify a motif from 400 to 423 amino acids (24 amino acids long), involving aa419, in the H protein of PPRV, which carries a CTL epitope, and is highly conserved among morbilliviruses, especially PPRV and RPV[47]. In addition, Renukaradhya *et al*. also mapped the H protein carrying B cell epitopes using mAbs for the presence of immuno-dominant regions, which suggested that the regions from 538–609 amino acids, including in aa574, are immunoreactive [12]. Amino acids 575–583 domain is unique to the RPV H protein and is an immuno-dominant epitope[48]. Amino acids 575 on RPV H protein is arginine. Interestingly, amino acid 575 on most PPRV H protein is also arginine. We believe that aa574 may also be an important position in the epitope of H protein. These amino acid changes at positive selection sites may confer a disadvantage to PPRV vaccine protection. So far, there has been no relevant report about aa176. Bewilderingly, the common site (aa246) of positive selection estimated by both methods is yet to be unraveled. It is unclear whether this site has any relationship with the interaction between the virus and host receptor or is a result of host specificity. Nonetheless, our research about the interaction between H protein and SLAM showed that the interface does not contain the common site. Our study indicated that amino acid change at positively selection sites did not lead to changes in the receptor of PPRV. All the positive selection sites were mapped on the H protein structure (Fig 2b).

**Fig 4:**
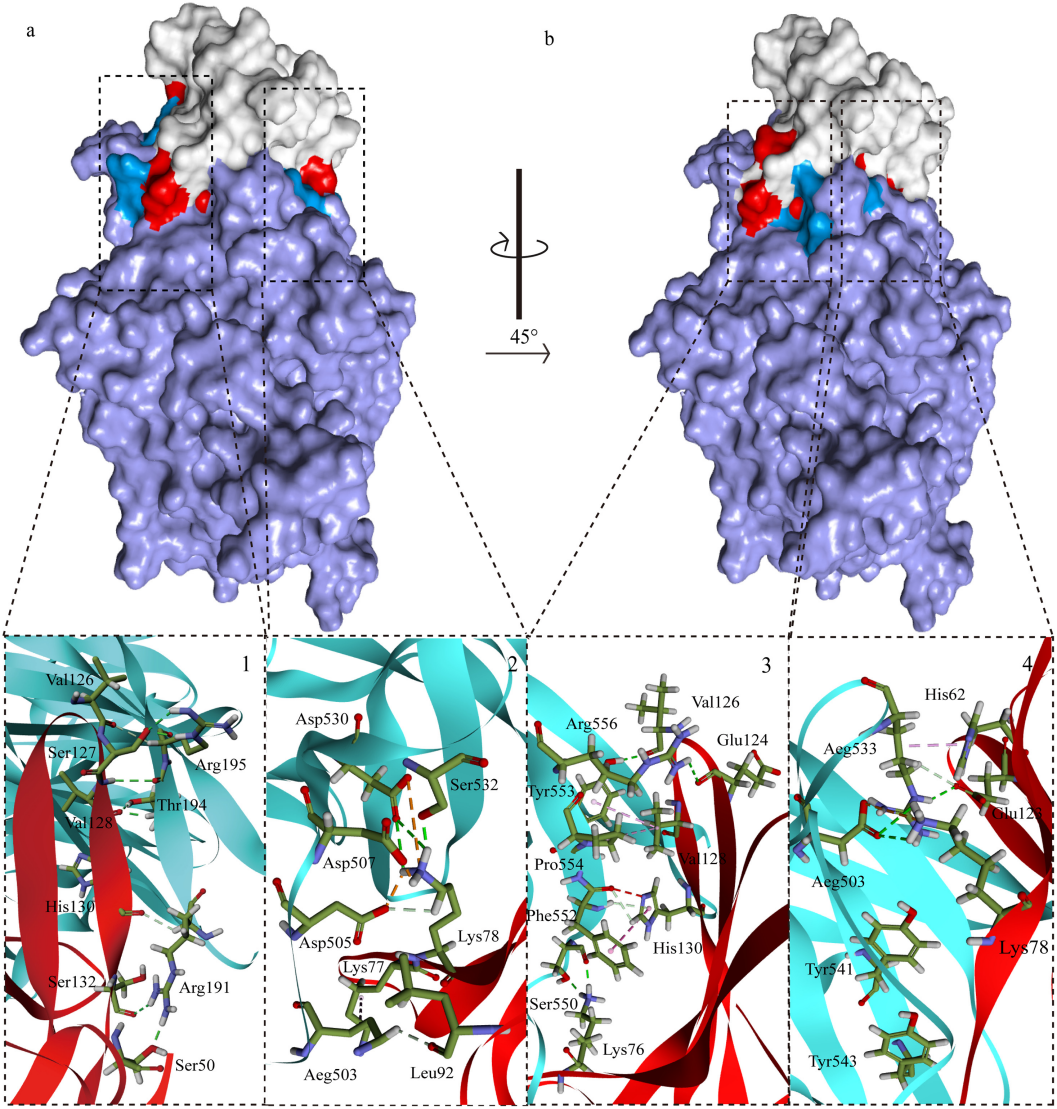
Solvent and stick drawing of the AAs from the interaction interface. The complex model shows four small regions of the binding interface between PPRV H (blue) and sheep SLAM (red) complex.

The structure of the measles virus hemagglutinin was reported by Christopher’s and Yusuke’s groups in 2007 [27,28]. The fold of MVH is a β-propeller with six blades (B1~B6) surrounding a large cavity, which is similar to our predicted structural model. Every one of blade contained four anti-parallel β-strands (S1–S4), and the six blades were connected through the loops between S1 of the next and S4 of one module [27]. In 2011, Yusuke’s group presented crystal structure of MVH-maSLAMV complex (MV H and the V-set Ig domains of marmoset SLAM)[49]. The crystal structure suggested that there were four small areas (sites 1–4) of the binding interface between MVH and SLAM. Our results show that the groove in the B4 blade and B5 of the head domain binds to the AGFCC′ β-sheets of the membrane-distal ectodomain of shSLAM, which the C β-sheet, C′β-sheet, and the loop of the two β-sheets are core regions of the interface. From the analysis of acting force, hydrogen bond, and hydrophobic force are the maintenance forces. There is an abundant presence of hydrogen bonds, electrostatic interactions and hydrophobic interactions in the interface of PPRVHv-shSLAM. The complex model showed four small regions of the binding interface (Fig 4): 1) is an intermolecular β-sheet assembled by the polypeptide backbones of the Arg191–Arg195 strand of PPRV Hv and the Val126–Ser132 strand of shSLAM, and there is a hydrogen bond in Arg191 of Hv and Ser50 of SLAM; 2) is formed by salt bridges of Asp505 and Asp507 in the proposed acidic patch of PPRV Hv to Lys78 of shSLAM. In addition, there are two hydrophobic interactions of PPRVHv Arg503 with shSLAM Lys77 and Leu92; 3) is formed by Pi interactions of PPRVH Phe552 to Pro554 and shSLAM Val128 and His130 as well as some hydrophobic interactions of PPRVH Ser550, Phe552, Tys553, Pro554, and Arg556 with shSLAMLys76, His130, Val128, Glu124, and Val126; and 4) are aromatic stacking of Tyr541 and Tyr543 of PPRV H with Phe119, and Pi interaction of Arg533 with His62 of shSLAM, and hydrogen bonds of PPRV H Asp530 and Arg533 with Lys78 and Glu123 of shSLAM. Comparing the interaction energy of the protein-ligand, we found out that mechanism of interaction of both the viruses and the receptors were very analogous, and interaction energy was also similar. Our studies indicate PPRVHv-shSLAM binding interface and MVH-maSLAM binding interface were consistent. Phe552, Arg533, Arg191, Tyr543, Arg503, Asp505 and Asp507 on PPRVHv; and Lys78, Lys76, His130, His62, Leu64, Val128 and Glu123 on shSLAM play a key role in the PPRVHv-shSLAM interaction.

Recently, Yusuke’s group used crystal structures of MV-H–receptor complexes and functional studies to reveal the mechanism behind the long-term success of the measles vaccine *via*[50]. The crystal structures of MVH–SLAM and MVH-CD46 complexes showed that both receptors target an exposed small region, and mutagenesis studies demonstrated that Nectin-4 binds to this area. The above studies proved that this region is the compact domain of the epitope. Therefore, it is of great importance to study whether those sites play the crucial role in pathogenicity, interspecies transmission, or immunoreaction, which may contribute in designing novel vaccines against PPRV for elimination of the disease.

## Supporting Information

S1 Table Virtual mutation of Hv residues from the interface of PPRVHv-shSLAM complex. * Energy unit = kcal.
(DOCX)

S2 Table Virtual mutation of sheep SLAM residues from the interface of PPRVHv-shSLAM complex. * Energy unit = kcal.
(DOCX)
