# Supplementary material for: Molecular Evolution and Characterization of Hemagglutinin (H) in Peste des Petits Ruminants Virus
Source: PLoS One. 2016 Apr 1;11(4):e0152587. doi: 10.1371/journal.pone.0152587 (PMC4818033; doi:10.1371/journal.pone.0152587)
Supplement: S1 Table — * Energy unit = kcal. (DOCX) [file pone.0152587.s001.docx]

**S1 Table.** **Virtual mutation of Hv residues from the interface of PPRVHv-shSLAM complex**

| **Mutation** | **Mutation Energy** | **Effect of Mutation** | **VDW Term** | **Electrostatic Term** | **Entropy Term** |
| --- | --- | --- | --- | --- | --- |
| LEU189 | 0.14 | NEUTRAL | 0.31 | -0.02 | 0 |
| GLY190 | -0.08 | NEUTRAL | -0.14 | -0.01 | 0 |
| ARG191 | 1.92 | DESTABILIZING | 7.23 | -0.1 | -2.06 |
| THR192 | -0.1 | NEUTRAL | 0.31 | 0.01 | -0.32 |
| VAL193 | 0.15 | NEUTRAL | 0.64 | -0.01 | -0.21 |
| THR194 | 0.58 | DESTABILIZING | 1.02 | 0.15 | 0 |
| ARG195 | 0.71 | DESTABILIZING | 2.52 | 0.29 | -0.87 |
| ALA196 | 0 | NEUTRAL | 0 | 0 | 0 |
| LEU464 | 0.27 | NEUTRAL | 1.08 | -0.02 | -0.32 |
| ILE482 | 0.35 | NEUTRAL | 0.81 | -0.06 | -3.00E-02 |
| LEU483 | 0.84 | DESTABILIZING | 2.03 | -0.31 | -3.00E-02 |
| ILE498 | 0.27 | NEUTRAL | 0.65 | -0.09 | -1.00E-02 |
| ARG503 | 1.46 | DESTABILIZING | 5.35 | -0.05 | -1.49 |
| ASP505 | 1.14 | DESTABILIZING | 2.49 | -0.18 | -2.00E-02 |
| ASP506 | 0.1 | NEUTRAL | 0.47 | -0.36 | 5.00E-02 |
| ASP507 | 0.88 | DESTABILIZING | 0.94 | 0.42 | 0.25 |
| TYR524 | 0.57 | DESTABILIZING | 0.7 | -0.17 | 0.38 |
| ASP530 | 0.88 | DESTABILIZING | 0.76 | 0.69 | 0.19 |
| SER532 | 0.09 | NEUTRAL | -5.00E-02 | 0.57 | -0.21 |
| ARG533 | 2.49 | DESTABILIZING | 4.58 | 0.35 | 3.00E-02 |
| SER534 | -0.48 | NEUTRAL | 0.14 | -0.08 | -0.64 |
| TYR541 | 1.03 | DESTABILIZING | 1.78 | -0.09 | 0.23 |
| TYR543 | 1.51 | DESTABILIZING | 3.24 | -0.33 | 7.00E-02 |
| SER548 | -0.05 | NEUTRAL | 0.27 | 0.07 | -0.27 |
| SER550 | 0.57 | DESTABILIZING | 0.32 | 0.78 | 3.00E-02 |
| TYR551 | 0.47 | NEUTRAL | 0.72 | 0.1 | 7.00E-02 |
| PHE552 | 2.65 | DESTABILIZING | 5.54 | -0.04 | -0.12 |
| TYR553 | 1.74 | DESTABILIZING | 3.59 | -0.03 | -5.00E-02 |
| PRO554 | 1.11 | DESTABILIZING | 1.82 | 0.04 | 0.22 |
| ARG556 | 0.98 | DESTABILIZING | 3.7 | -0.27 | -0.92 |

* Energy unit = kcal
