# Supplementary material for: Molecular Evolution and Characterization of Hemagglutinin (H) in Peste des Petits Ruminants Virus
Source: PLoS One. 2016 Apr 1;11(4):e0152587. doi: 10.1371/journal.pone.0152587 (PMC4818033; doi:10.1371/journal.pone.0152587)
Supplement: S2 Table — * Energy unit = kcal. (DOCX) [file pone.0152587.s002.docx]

**S2 Table.** **Virtual mutation of sheep SLAM residues from the interface of PPRVHv-shSLAM complex**

| **Mutation** | **Mutation Energy** | **Effect of Mutation** | **VDW Term** | **Electrostatic Term** | **Entropy Term** |
| --- | --- | --- | --- | --- | --- |
| SER32 | 0.04 | NEUTRAL | 7.00E-02 | 0 | 0 |
| LEU48 | 0.04 | NEUTRAL | 8.00E-02 | -0.01 | 0 |
| SER50 | 0.23 | NEUTRAL | 1.00E-02 | 0.42 | 2.00E-02 |
| ILE61 | 0.36 | NEUTRAL | 1.3 | -0.05 | -0.33 |
| HIS62 | 1.56 | DESTABILIZING | 2.74 | 0.05 | 0.2 |
| LEU64 | 1.36 | DESTABILIZING | 2.35 | -0.1 | 0.3 |
| THR66 | 0.55 | DESTABILIZING | 0.92 | 0.02 | 0.1 |
| ASP73 | 0.24 | NEUTRAL | 3.02 | -1.81 | -0.46 |
| THR74 | 0.59 | DESTABILIZING | 1.03 | 0.03 | 7.00E-02 |
| VAL75 | 0.55 | DESTABILIZING | 1.36 | -0.01 | -0.16 |
| LYS76 | 2.47 | DESTABILIZING | 4.08 | 1.5 | -0.4 |
| LYS77 | 0.95 | DESTABILIZING | 3.2 | 0.8 | -1.31 |
| LYS78 | 4.87 | DESTABILIZING | 5.2 | 4.19 | 0.22 |
| SER81 | -0.56 | STABILIZING | -0.84 | -0.29 | 1.00E-02 |
| LEU92 | 1.38 | DESTABILIZING | 3.61 | -0.44 | -0.26 |
| SER121 | 0.09 | NEUTRAL | 0.15 | -0.11 | 9.00E-02 |
| GLU123 | 1.19 | DESTABILIZING | 3.17 | -0.94 | 9.00E-02 |
| GLU124 | -0.19 | NEUTRAL | 0.11 | -0.49 | 0 |
| ASN125 | 0.13 | NEUTRAL | 0.45 | -0.2 | 1.00E-02 |
| VAL126 | 0.71 | DESTABILIZING | 1.66 | 0.01 | -0.16 |
| SER127 | -0.3 | NEUTRAL | 1.02 | 0.07 | -1.06 |
| VAL128 | 1.23 | DESTABILIZING | 2.36 | 0 | 6.00E-02 |
| GLN129 | -0.3 | NEUTRAL | 0.83 | -0.11 | -0.83 |
| HIS130 | 1.83 | DESTABILIZING | 4.43 | -0.25 | -0.33 |

* Energy unit = kcal
